# A descriptive case report of telesupervision and online case-based learning for speech and language therapy students in Vietnam during the COVID-19 pandemic

**DOI:** 10.4102/sajcd.v69i2.897

**Published:** 2022-08-15

**Authors:** Lindy L. McAllister, Marie Atherton, Alison Winkworth, Stephanie Wells, Dien K. Le, Karina Sandweg, Thuy T.T. Nguyen, Natalia Henderson-Faranda, Sharon Broadmore

**Affiliations:** 1 Faculty of Medicine and Health, University of Sydney, Sydney, Australia; 2 Department of Speech Pathology, Faculty of Health Sciences, Australian Catholic University, Melbourne, Australia; 3 Private, Albury, Australia; 4 Private, London, United Kingdom; 5 An Bình Hospital, Ho Chi Minh City, Viet Nam; 6 College of Education, Health and Human Development, University of Canterbury, Christchurch, New Zealand; 7 Medical Committee Netherlands Vietnam (MCNV), Hanoi, Vietnam; 8 Natalia HF Speech & Language Therapy, Christchurch, New Zealand; 9 Kinela Speech Therapy, Melbourne, Australia

**Keywords:** COVID-19, pandemic, low- and middle-income countries, speech and language therapy, education, clinical placement, online learning, case-based learning, telesupervision

## Abstract

**Background:**

Vietnam’s first speech and language therapy (SLT) degrees commenced in 2019 utilising international educators. Continuity of the degrees was impacted by travel restrictions during the coronavirus disease 2019 (COVID-19) pandemic.

**Objectives:**

This article presents a descriptive case report exploring the viability of online learning to continue clinical education (CE) of SLT students in Vietnam during the pandemic.

**Method:**

Students were scheduled for face to face placements throughout 2021. International SLT educators were to travel to Vietnam and work with interpreters and locally trained certificate level therapists to provide placement supervision. When travel became impossible, tele-supervision by international therapists working remotely and in partnership with local therapists and interpreters was arranged. The second wave of Covid-19 excluded students from healthcare settings early in their placements. To conclude these placements, tele-supervisors led online case-based discussions with students. For subsequent placements, Vietnamese and international therapists facilitated two to three weeks of online case-based group discussions for students, using cases with videos or avatars.

**Results:**

Learning outcomes for students, as evidenced in written and oral assessments demonstrated attainment of many of the learning objectives of the placements. Satisfaction for all participants (students, tele-supervisors, online group facilitators) was high. Students will undertake face to face placements in the future; however they will commence these placements with heightened clinical reasoning and planning skills.

**Conclusion:**

Online CE is possible in LMIC and, as part of a program which includes face to face placements, can support essential CE outcomes and enhance preparation for subsequent direct experiences with patients.

## Introduction

Cessation of face-to-face teaching because of coronavirus disease 2019 (COVID-19) pandemic lockdowns meant a rapid pivot for many health profession degrees to online learning, including telehealth placements, with or without telesupervision (TS). Lyons et al. (2021), for example, described the rapid development of a telehealth service in Ireland to enable student placements, whilst Kamper, Dario, Waibel, O’Connor and Bourne (2021) described the rapid repurposing of existing telehealth services to include student placements during the pandemic. Other programmes in high-income countries (HICs) used online case-based learning (CBL) and/or online simulation-based learning (SBL) (see, e.g. Taylor & Salmon, 2021). This pivot was possible in many HICs where information and communications technology (ICT) device ownership is high and there is access to high-speed Internet with sufficient bandwidth to support online learning; it was also made possible by many universities and affiliate health care facilities having existing telehealth services, teleplacements and TS programmes, CBL or SBL programmes or the capacity to rapidly develop or acquire cases for online learning (see, e.g. May, Grotowski, Walker, & Kelly, 2021).

To date, few studies have addressed the use of online learning in health degrees in low- and middle-income countries (LMICs) during the pandemic. Li et al. (2021) reported a range of barriers and facilitators to online medical and nursing education in China during the pandemic. In discussing occupational therapy placements in South Africa during the pandemic, Gurayah (2021) reported challenges including the cost of data, student access to laptops and learning how to use virtual conferencing and online learning platforms. The present article adds to the small body of literature about the use of online learning in LMICs by describing the use of TS and online CBL for speech and language therapy (SLT) students in Vietnam during the COVID-19 pandemic.

Telesupervision is defined as ‘the use of ICT for communication between university-based staff, clinical supervisors and/or students undertaking placements in the presence or absence of a clinical supervisor onsite’ (Nagarajan et al., 2016, p. 18). High-quality TS requires the management of technology-related issues, including access to equipment, Internet, good bandwidth, data security and confidentiality and ethical considerations such as patient consent and privacy (see Nagarajan et al., 2018). McCabe, Purcell, Baker, Madill and Trembath (2009, p. 309) defined CBL as ‘the use of real-life or hypothetical clinical cases to promote student thinking and learning in ways that simulate clinical practice’. Various researchers have reported good learning outcomes for students using TS (see, e.g. Lyons et al., 2021; Nagarajan et al., 2016; Overby, 2018; Taylor & Salmon 2021) and for CBL (McCabe et al., 2009). In Vietnam, online learning and simulation were rarely used in university programmes prior to the pandemic; therefore, banks of cases for conversion to online CBL were not available. Students mostly had smart phones but not necessarily laptops, and Internet with good bandwidth was often limited in their homes. When the pandemic hit, two university-based SLT degrees were being run in Vietnam – 14 students were completing a two-year Master in SLT (MSLT) at University of Medicine and Pharmacy in Ho Chi Minh City, and 20 students were completing a four-year Bachelor in SLT (BSLT) at Da Nang University of Medical Technology and Pharmacy. Development and delivery of these degrees were undertaken through a multilateral international partnership between two nongovernmental organisations: the Vietnam-based Medical Committee Netherlands Vietnam (MCNV) (https://mcnv.org/) under its SLT Education Development Project, funded by U.S. Agency for International Development (USAID) through VietHealth, with the Australia-based Trinh Foundation Australia (TFA) (https://trinhfoundation.org/) providing professional input. The students had completed one or two of six planned face-to-face placements before the pandemic lockdowns halted all placements and classroom-based learning. This article’s focus is on the perspectives of Vietnamese and international clinical educators involved in the two degree programmes, although it is acknowledged that the perspectives of students are also important and under-researched (Lyons et al., 2021; Overby, 2018).

## Methods – how telesupervision and online case-based learning were used

### Telesupervision

**FIGURE 1:**
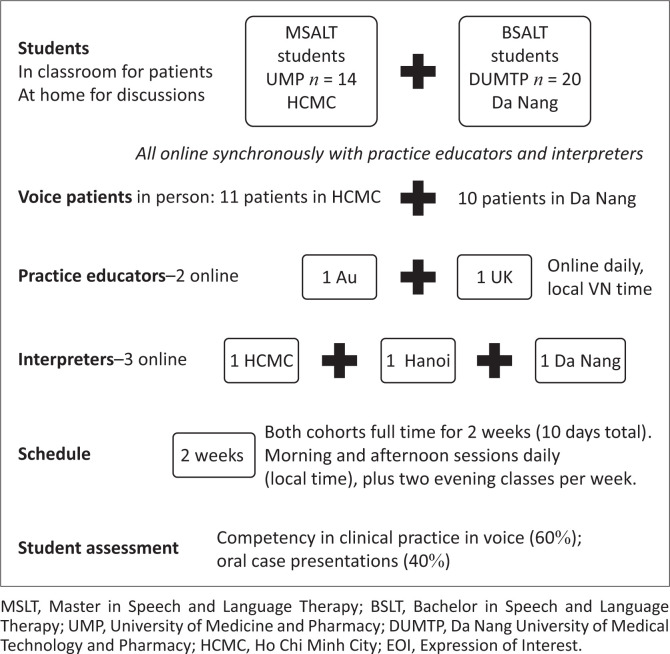
CP2 Voice: real patients in classroom with students, educators and interpreters online.

The first TS of a clinical placement (CP2) was a two-week (ten-day) intensive course for both MSLT and BSLT cohorts simultaneously, working with voice patients in the two university SLT teaching clinics with two telesupervisors: one in Australia and one in the United Kingdom. Three SLT-trained interpreters living in Vietnam interpreted online in shifts during the two weeks. Facilitated by the telesupervisors and supported with interpreters, students not only participated in sessions directly with patients in the university clinics but also observed each other’s sessions and came together via Zoom for facilitated planning and debriefing discussions. Figure 1 illustrates key inclusions in the placement.

Supported by written teaching materials translated in advance, the schedule for the two weeks included refresher learning activities in the first two days, followed by six days of patient sessions intermingled with discussions, debriefing and planning to guide clinical reasoning. The schedule was deliberately designed to include facilitated discussions before and after all patient sessions, with a focus on clinical reasoning. Students were assessed by the telesupervisors for clinical competence in voice (60% of grade) and their individual (MSLT) or paired (BSLT) case presentations (40%) conducted over the last two days of the placement.

The synchronous online sessions occurred on the Zoom platform, and there were various technical difficulties, mostly because of intermittent poor audio or video quality. Despite these difficulties, the rollout of the subject was successful, especially considering the complexity and logistics of managing two Zoom accounts, using microphones for amplification in the classrooms, telesupervisors each in different time zones, three interpreters in different shifts and locations and students together in two university clinics for patient sessions, then at their home or work later for facilitated discussion sessions. Prerecorded videos of telesupervisors working with patients were made as backup in case patients were unable to attend at short notice. These did not have to be used, as all patients attended as planned.

Telesupervision was also planned for the penultimate and final placements in both degrees. Students were to be placed in Vietnamese hospitals, with patients organised by Vietnamese speech and language therapists (hereafter therapists) in those facilities. Workloads of Vietnamese therapists meant they could not manage supervision of students and their normal clinical work simultaneously, and so international therapists were recruited and trained to provide online TS, using remote viewing of student interactions with consenting patients or families. However, in the weeks prior to the first TS placement for MSLT students, some hospitals advised they would not allow remote viewing of patients by TS, even though patients had consented to this. Where allowed, tablets were used to transmit a student’s session with a patient, and the interpreter typed on her computer, translating live. The telesupervisor provided suggestions for the student through the interpreter in real time. Unfortunately, within the first week of the TS placement, the second wave of the pandemic meant students were excluded from these and all subsequent placements.

### Online case-based learning

The inability to continue face-to-face contact with patients during placement via TS required a rapid switch by the telesupervisors to online CBL. The goals of the online CBL for both the MSLT and BSLT placements were to develop students’:

1. ability to identify key diagnostic and other relevant information about a patient’s communication and/or swallowing difficulties (CSwD)
2. knowledge of assessment and intervention options for a range of patients with CSwD
3. skills in planning culturally appropriate assessment and intervention sessions
4. clinical reasoning ability and their capacity to articulate this, with reference to the evidence-base, the International Classification of Functioning, Disability and Health (World Health Organization, 2001) and cultural considerations.

For the first week of the terminated placement, the international telesupervisors provided patient cases from their clinical experiences for online discussion with students. Interpreters were present for all discussions. Patient information, assessment priorities, tools and findings were discussed, and management priorities and strategies were identified. Differences were also explored regarding how patients might be managed in Vietnam versus the HIC where the telesupervisors were based, with a focus on identifying culturally and contextually relevant management objectives, resources and supports.

Given the time-intensive nature of developing multiple culturally relevant cases for discussion, licences were purchased for each student and CBL group leader for a commercially available, English language CBL platform comprising patient documentation, videos and avatars. The platform was considered to offer a more ‘authentic’ experience for students than paper-based CBL alone. Further, the platform provided extensive case notes about patient presentations, social and contextual information, assessment and treatment.

Cases were selected from the platform to illustrate a variety of paediatric and adult CSwD arising across the lifespan. For the paediatric placement, cases related to children with complex disabilities (e.g. cerebral palsy, autism spectrum disorder, Down syndrome, traumatic brain injury [TBI] and hearing loss); for adult placements, cases illustrated CSwD arising from stroke, motor neurone disease, head and neck cancer and TBI. We were aware that cases from an HIC might be limited in terms contextual and cultural appropriateness and relevance. Therefore, Vietnamese authors on the present article reviewed cases, selected those that were contextually relevant and provided advice around cases and materials that required modification to reflect the local context. Selected cases were typical of patients seen in Vietnamese health care settings. Summaries of information relevant to each case were prepared in Vietnamese for the students. Case-based discussion with the students included time for critique of which assessment and treatment approaches used in the case study were relevant to the Vietnamese context and how assessments and interventions for this case could be made more culturally and contextually appropriate.

Students and CBL group leaders worked through Zoom from their homes using personal laptops or phones. Both local Vietnamese and non-Vietnamese international therapists led the discussions, with an interpreter present for the non-Vietnamese-speaking group leaders. Groups generally comprised one CBL group leader and four to six students. When larger groups of students were necessary, students were allocated into smaller groups in Zoom breakout rooms, and the group leader rotated through the rooms to support student discussions before reconvening the whole cohort. The duration of the group sessions was generally 2–3 hours per day for up to 10 hours per week over 4–5 weeks.

#### Format of case-based learning discussion sessions

Case-based learning group leaders guided students through nominated cases and associated learning materials and tasks, set activities for students to complete between group meetings and provided feedback between sessions. Some of the activities were determined by the learning materials for each case, but activities that addressed discussion points raised in group meetings were also set. Group discussions supported all students to be involved and encouraged open debate, both between students and between students and group leaders. As a means of debriefing and ensuring as equitable an experience as possible for all students, the group leaders met via Zoom at the end of each week to discuss their experiences and plan for the following week.

Ethical approval to report telesupervisor and CBL facilitator perspectives was not required, as the information was collected as part of a routine teaching evaluation.

### Ethical considerations

As information presented in this article was collected as part of routine teaching evaluation, ethics approval was not required.

## Results – What we found

### Challenges encountered and strategies used to manage these

We expected to manage challenges using TS and CBL. These included communicating through interpreters, intermittent and various difficulties with Internet connection and some difficulties determining student engagement. Forward planning was essential for scheduling and working with interpreters, including translation and editing of written materials well in advance. Furthermore, in real-time online Zoom sessions, the use of the text-based chat box assisted all parties (educators, interpreters and students) as it provided written text that could be tracked, in addition to viva voce simultaneous interpreting.

With stakeholders in various locations and different time zones, patchy Internet access and bandwidth were to be expected, especially with sessions that took place whilst students logged in from their homes instead of the classroom. At times, it was difficult to identify whether students had Internet connection issues or were experiencing difficulty engaging with the learning activities and discussions. Students were all encouraged to write their questions down, either in the chat box for immediate translation where possible or for submission to the interpreter for translation to the educator who could answer them in the next discussion session. Table 1 summarises these challenges and strategies.

**TABLE 1:** Challenges encountered and strategies used.

Challenges	Strategies
**Using interpreters**	
Long lead times for translating written materials Few expert interpreters, full schedule Simultaneous interpretation lengthens all live communication	Plan months in advance, succinct written materials. Use same interpreters for live teaching. Agreed schedule between all stakeholders, identify a larger pool of interpreters. Less experienced interpreters observe and participate. ‘Understudy’ programme and backup plans. Use the chat box function in the live online platform so any on-the-spot questions and answers can be translated more easily.
**Facilitators and educators**	
Sourcing facilitators with relevant experience Some facilitators had no previous experience working in Vietnam Clarifying the respective roles (e.g. of local supervisors and international facilitators)	Wide-ranging call for expressions of interest (EOI) via various professional speech and language therapy groups. Comprehensive briefing and training and planning for peer- and expert-level support. Explicitly clarify responsibilities of various stakeholders. Set up regular meeting times between local and international supervisors for debriefing and planning.
**Internet issues**	
Managing complexity, given stakeholders in numerous locations Intermittently poor connections	Clear schedule communicated in advance: when students were required in classroom versus allowed to work from home (WFH). Students appreciate flexibility in WFH where possible. Maximise flexibility when Internet is poor, provide post-session debriefing and discussion when reconnected.
**Determining student engagement**	
Managing the dynamics of group learning online Difficulty discriminating between apparent Internet access issues and possible lack of individual engagement	Plan small group or paired activities where possible. Clarify expectations for how sessions will be conducted. Specify learning activities that require student responses. Structure session sequence and progression to prepare students for increased responsibility. Facilitators provide feedback to the group(s).

For TS and CBL or SBL, it was also critical to recruit educators who had relevant experience or provide training ahead of time. For CBL or SBL specifically, it was important to develop culturally and locally relevant cases of appropriate complexity for the student cohort.

### Learning outcomes for students

Learning outcomes for students in all placement subjects in which TS or CBL was used demonstrated attainment of many of the placement learning objectives written in subject outlines before the pandemic. Assessments in the telesupervised CP2 placement showed that all students met expected learning outcomes for assessment and treatment for common voice disorders, and most students achieved high case presentation scores. Students’ written reflections based on the requirement to observe all patient sessions when they were not involved in direct patient care demonstrated growth in clinical knowledge as well as increased awareness of their own progress towards competency.

Assessment of students for the CBL involved written case studies and individual and small group presentations of cases and associated oral exams. Assessments followed a developmental trajectory in that over the duration of the placements, students were required to demonstrate increasing ability to identify, analyse and synthesise relevant information, consider holistic and person-centred approaches to assessment and management and tailor these to the Vietnamese context.

For the first MSLT CBL placement, nine students passed in their first attempt, with five students successful on a resit. These five students received additional input that included detailed written and verbal feedback and tutorial support to improve the content and delivery of their presentations. On subsequent CBL placements, all MSLT students and BSLT students passed written case studies and/or small group case presentations and oral vivas in the first attempt.

### Experiences of telesupervisors and online case-based learning group leaders

The experience of online teaching was challenging for the telesupervisors and CBL group leaders. Internet reliability and signal dropout were not uncommon for students, interpreters and educators. Facilitating critique of the CBL cases for contextual and cultural relevance was challenging for non-Vietnamese CBL group facilitators, who sought advice from their Vietnamese colleagues also involved in CBL groups. Despite having interpreters trained in SLT terminology, the open-ended nature of discussions sometimes meant interpreters encountered unfamiliar English vocabulary and would need time to develop an appropriate interpretation. In addition, interpreters were occasionally unavailable for scheduled classes because of personal health issues. Furthermore, the online environment, with cameras sometimes off to reduce draw on bandwidth and avoid dropout, meant that the telesupervisors and CBL group leaders found it difficult to gauge students’ levels of engagement and respond appropriately.

Overall, however, the telesupervisors and CBL leaders reported the challenges of teaching online were managed as well as possible and that good learning outcomes were achieved for students. All expressed satisfaction with their contributions and indicated willingness to stay involved in educating the Vietnamese SLT students in the future. They found online teaching to be an ‘invaluable experience’, providing comments such as ‘wonderful to see students gain confidence with online supervision and case discussion’, and ‘students generally well prepared and engaged; brought interesting cases for discussion’. They found in particular that most students were able to solve problems with sound clinical reasoning. A well-experienced Vietnamese SLT who acted as a CBL group leader reported that ‘the varied and well-structured cases favourably assisted the students for [face to face] placement later’.

## Summary and conclusions

The pandemic has caused many disruptions and challenges for university education of health professionals globally, particularly for LMICs, but the use of ICT to facilitate synchronous online learning offers a practical solution. The use of ICT and innovative educational design for TS and CBL for SLT education in LMIC in this case has meant that students’ CE was progressed and extension of courses averted. This was critical given funding rules and timelines for the project. Although ICT and language differences created communication issues, these were not insurmountable, and students had access to powerful learning experiences with expert guidance and facilitation in real time, including practice with voice patients who also benefited from the students’ and educators’ skills.

On reflection, what was a rapid pivot to enable ongoing CE for SLT students in Vietnam has provided numerous lessons for embedding CBL and TS in future SLT education in Vietnam. Online CBL strengthened students’ clinical reasoning skills, evident in subsequent face-to-face placements. Therefore, we plan to include CBL (either online or face-to-face) as preparation for placements in future SLT degrees in Vietnam. Ideally, Vietnamese speech and language therapists should begin to develop banks of culturally and contextually appropriate cases, reducing both the need for and cost of commercial banks of cases which still require modification. We have also learnt that there should be a backup interpreter available at all times should availability issues arise because of Internet or personal challenges for scheduled interpreters. The communication issues because of language differences which we encountered will naturally reduce as the growing number of Vietnamese therapists take over education of SLT students in Vietnam from international SLTs participating in the initial degrees. However, TS will remain as an option to support local Vietnamese therapists to develop expertise in CE and supervision. We believe that the methods reported in this article and possibilities for embedding in future degrees will be of interest to other SLT degrees in LMICs.

In this article, we have not focused on the students’ experiences of online learning, but their views will be gathered as part of a large-scale evaluation of their courses, with a view to future reporting. Online learning is not a full replacement for real placement experience, and therefore we are planning for clinical placements for both cohorts of students as soon as the pandemic allows the resumption of face-to-face placements and supervision.
